# White matter conduction in the human brain is mostly slow, with rare high velocity connections

**DOI:** 10.64898/2026.07.28.741279

**Published:** 2026-07-30

**Authors:** Samuel Romero-Santiago, María Guadalupe Yáñez-Ramos, Jordan A. Bilderbeek, Nicholas M. Gregg, Myung-Ho In, Erin Gray, Daehun Kang, Yunhong Shu, Gregory A. Worrell, Cameron C. Mcintyre, Kai J. Miller, Dora Hermes

## Abstract

White matter bundles play a crucial role in cognitive functions by rapidly transmitting information between brain regions. Inter-areal conduction guides the integration of information and conduction velocity is a fundamental parameter in theories and models of brain function. However, distributions of conduction velocity remain difficult to characterize in vivo in humans. In this work, we integrated diffusion magnetic resonance imaging (dMRI) tractography with intracranial electrical stimulation during clinical stereo-electroencephalography (sEEG) monitoring in 17 subjects to measure conduction velocity within four major white matter bundles. Our findings reveal that human brain conduction is characterized by high variability both within and between bundles, reflecting a predominance of slow connections alongside rare high-speed connections. Because conduction velocity in myelinated fibers follows an approximately linear relationship with axon diameter, we derive underlying axon diameter distributions and show that these estimates are comparable to previous post-mortem studies. These findings demonstrate a heavily skewed distribution of human neural conduction velocities and show that structural heterogeneity shapes the timing and integration of information in large-scale networks.

## Full Text Availability

The license terms selected by the author(s) for this preprint version do not permit archiving in PMC. The full text is available from the preprint server.

